# Global validation of data-assimilative electron ring current nowcast for space weather applications

**DOI:** 10.1038/s41598-024-52187-0

**Published:** 2024-01-28

**Authors:** Bernhard Haas, Yuri Y. Shprits, Michael Wutzig, Mátyás Szabó-Roberts, Marina García Peñaranda, Angelica M. Castillo Tibocha, Julia Himmelsbach, Dedong Wang, Yoshizumi Miyoshi, Satoshi Kasahara, Kunihiro Keika, Shoichiro Yokota, Iku Shinohara, Tomo Hori

**Affiliations:** 1 GFZ German Research Centre for Geosciences, Helmholtz Centre Potsdam, Potsdam, Germany; 2 Institute of Physics and Astronomy, University of Potsdam, Potsdam, Germany; 3 Department of the Earth, Planetary and Space Sciences, University of California, Los Angeles, CA USA; 4 ISEE, Nagoya University, Nagoya, Japan; 5 School of Science, University of Tokyo, Tokyo, Japan; 6 Osaka University, Toyonaka, Japan; 7 Japanese Aerospace Exploration Agency, Tokyo, Japan

**Keywords:** Magnetospheric physics, Magnetospheric physics

## Abstract

The hazardous plasma environment surrounding Earth poses risks to satellites due to internal charging and surface charging effects. Accurate predictions of these risks are crucial for minimizing damage and preparing for system failures of satellites. To forecast the plasma environment, it is essential to know the current state of the system, as the accuracy of the forecast depends on the accuracy of the initial condition of the forecast. In this study, we use data assimilation techniques to combine observational data and model predictions, and present the first global validation of a data-assimilative electron ring current nowcast during a geomagnetic storm. By assimilating measurements from one satellite and validating the results against another satellite in a different magnetic local time sector, we assess the global response and effectiveness of the data assimilation technique for space weather applications. Using this method, we found that the simulation accuracy can be drastically improved at times when observations are available while eliminating almost all of the bias previously present in the model. These findings contribute to the construction of improved operational models in estimating surface charging risks and providing realistic ’source’ populations for radiation belt simulations.

## Introduction

Satellites flying through near-Earth space are constantly affected by charged particles. This potentially hazardous environment can cause satellite anomalies through internal charging or surface charging effects^1–4^. As an increasing number of satellites are launched into space, accurate prediction of these risks becomes vital, as this enables satellite companies to minimize damage, and can prepare operators before system failures.

In order to accurately forecast the radiation belts and ring current forming this hazardous plasma environment, it is necessary to know the current state of the system. Surface charging effects are caused by lower energy electrons (10–50 keV)^3^, which are very dynamic, and therefore difficult to predict with physics-based codes alone. Despite these difficulties, several ring current models have been built to study the electron flux within the ring current in recent decades^5–7^. While these models are able to capture the basic dynamics of the ring current during geomagnetically quiet times, simulating highly dynamic geomagnetic storms still poses a formidable challenge. Nevertheless, ring current models aim to be operational, to forecast the surface charging environment and its risks for spacecraft in real-time^8^. In an operational framework, it is desirable that all available information about the system is incorporated into the model since this provides the most reliable solution possible. One great source of information is the utilization of real-time or near-real-time data streams of satellites flying through the ring current, as they are capable of directly observing the target variable, electron flux. Satellites at low Earth orbit (LEO), are also capable of generating large amounts of high cadence information about precipitating electrons, which could be useful to incorporate into ring current models.

Merging information given by measurements and models is called data assimilation. It has already proven to be a useful tool in meteorology, oceanography, navigation, and recently, also in space weather applications, such as ionosphere^9,10^ and radiation belts^11,12^ modeling.

The Kalman filter^13^, a widely used data assimilation algorithm we are also adopting in this study, is an optimal recursive estimator that sequentially updates the estimated state and its uncertainty based on both the available measurements and knowledge of the system dynamics. It provides a systematic framework for assimilating observational data, accounting for model errors, and refining the estimates of the model state. The Kalman filter propagates the state estimate using the underlying physics-based model while incorporating the measurements to refine the estimate in two main steps: prediction and update step. By iteratively repeating these steps, the Kalman filter dynamically adjusts the model state estimate, effectively assimilating the available measurements.

Recently, the Kalman filter has been used to study the applicability of data assimilation to ring current simulations^14,15^. Other data assimilation approaches have also been investigated, including direct insertion of satellite measurements^16^, a particle filter to assimilate global imaging data for energetic neutral atoms^17^, and ensemble Kalman filters^18^ for radiation belts modeling^19,20^. The first Kalman filter applied to the ring current, was an ensemble Kalman filter based on orthogonal projections to reduce the dimensionality of the problem^15^. In this study, ion flux measurements from one satellite have been assimilated into the ring current model, while validating the results against a satellite in the same magnetic local time (MLT) sector. The authors concluded that the particle injections during a substorm event have been correctly captured in the data-assimilative model, which greatly reduced the model errors compared to observations. Similarly, another study demonstrated an application of a log-normal Kalman filter^21,22^ to refine electron ring current simulations^14^. The authors performed synthetic twin experiments to show that the log-normal Kalman filter is able to correct an incorrect model state and converge to the synthetic data. Additionally, they assimilated in situ measurements from a single satellite and showed that the model predictions have been greatly improved when compared to the assimilated data. These studies prove that data assimilation techniques can be applied to electron and proton ring current simulations, and are able to improve model results locally. However, the global effect of data assimilation has not been investigated or properly validated on an independent data set. Moreover, it remains unclear if data assimilation techniques are capable of reconstructing the entire ring current at all MLT, or if they are only helpful for locally correcting the forecasted state of the system.

In this work, we present the first global validation of a data-assimilative electron ring current nowcast during a geomagnetic storm. We are assimilating measurements from one satellite while validating the results against another satellite in a different magnetic local time sector. This experiment setup allows us to study the global response of our model to the assimilated data and the technique’s effectiveness for space weather applications. Our results show that data assimilation can be used in operational models to estimate the risk of surface charging effects and to provide realistic ’source’ populations for radiation belt simulations.

## Results

### Observations during the geomagnetic storm occurring on September 7, 2017

**Figure 1:**
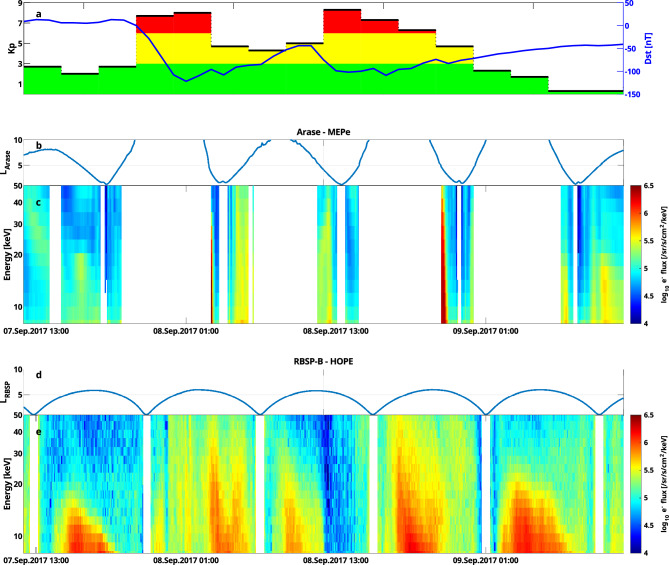
Geomagnetic indices and satellite data for the geomagnetic storm studied in this work. (**a**) The Kp and Dst time series. (**b**) L shell observed by Arase calculated using the T04s magnetic field model^31^. (**c**) Electron flux measurements taken by Arase interpolated to 30° equatorial pitch angle. (**d**) L-shell observed by RBSP-B. (**e**) Electron flux measurements taken by RBSP-B interpolated to 30° equatorial pitch angle.

In early September 2017, Earth’s magnetosphere was impacted by a series of coronal mass ejections (CME) and solar flares^23^. This triggered a strong geomagnetic storm, with a Kp maximum of 8 + and a Disturbed Storm Time (Dst) index minimum of -122 nT, which lasted for about 36 hours (see Fig. 1a). Various studies investigated the response of the ionosphere and thermosphere during this event^24,25^, while other studies investigated the large geomagnetically induced currents^26^ and radiation belt dynamics^27^ during this event.

**Figure 2:**
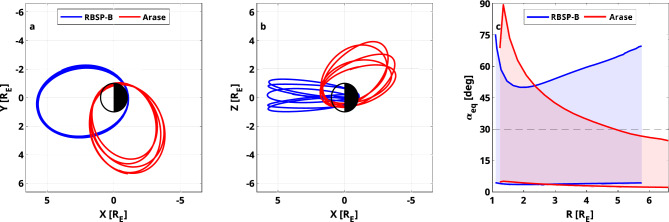
Orbits of satellites used in this study in GSM coordinates. (**a**) Orbit of the RBSP-B and Arase satellites projected to the equatorial plane. (**b**) Orbit of the satellites projected to the meridian plane. (**c**) Equatorial pitch angle coverage of the satellites as a function of radial distance.

We also chose this event for our data assimilation study, since during this time, two satellite missions designed for studying the ring current and radiation belts were operative simultaneously: NASA’s twin satellites, the Van Allen Probes^28^, and JAXA’s Arase^29,30^ satellite. Looking at the MLT distribution of the satellites (see Fig. 2a), the Van Allen Probes resided on the dayside during this event, while Arase measured the incoming electrons on the nightside. In addition, the highly elliptical orbit of both missions allows us to study the ring current across the entire inner magnetosphere up to almost geosynchronous orbit. Combining both data sets makes it possible to study the global response of the ring current during this event.

As we focus on the 10–50 keV energy range, we use measurements taken by the Helium, Oxygen, Proton and Electron (HOPE)^32^ instrument on board of the Van Allen Probes, and the Medium Energy Particle Experiment (MEPe)^33^ instrument from Arase. Due to the highly inclined orbit of Arase (see Fig. 2b), we compare electron flux at 30° equatorial pitch angle, where both satellites can observe particles (see Fig. 2c). Figure 1 shows the flux spectrogram of Arase and RBSP-B interpolated to 30° equatorial pitch angle along their measured L shell (the radial distance of the satellite position normalized by the Earth’s radius, when mapped to the equatorial plane) during their flybys. The displayed Arase data seems to be sparse since it does not observe electrons with 30° equatorial pitch angle above L = 5. While Arase is flying through the nightside, it occasionally measures the injection of electrons, visible as high fluxes during the main phase of the storm. RBSP-B, residing on the dayside, measures highly dynamic flux values, observing both depletions and enhancements throughout the event. Previous studies have shown, that measurements from the MEPe and MagEIS^34^ instruments agree well with each other^35,36^. By looking at physical conjunctions between Arase and the Van Allen Probes during their two-year overlap, we find that the HOPE and MEPe instruments agree extraordinarily well with each other as well (see Supplementary Figs. S1 and S2), allowing us to use both data sets together without correcting for any biases.

We choose to assimilate data obtained by Arase into our ring current model while validating against RBSP-B measurements, as Arase is capable of correcting the electron flux as soon as it enters on the nightside.

### Data-assimilative simulations of the storm event

The ring current model used in this work is based on the VERB-4D model, which is capable of describing the evolution of the electron phase space density in the inner magnetosphere in a convective-diffusive manner^37^. For lower energy electrons (< 50 keV), the diffusive terms may be neglected, as particles are dominated by convection, leading to a Convection-Simplified variant of VERB-4D called VERB-CS^14,38,39^. The model uses empirical models to specify the electric field, magnetic field, and flux at the outer radial boundary, which is chosen at geosynchronous orbit. The full model setup is described in the Methods section.

In this work, we use the log-normal Kalman filter as our data assimilation algorithm, which was specifically designed to preserve the fundamental positivity of phase space density^21,22^. The algorithm was previously applied to VERB-CS, where synthetic twin experiments showed the convergence and effectiveness of the algorithm^14^. The model has to be linearized in order to be used inside the Kalman filter, which means dropping the term describing the loss of particles and solving the convection equation using a 1st order scheme. The details of the linearization process, the data assimilation algorithm, and a description of the evolution of the covariances are provided in the Methods section.

**Figure 3:**
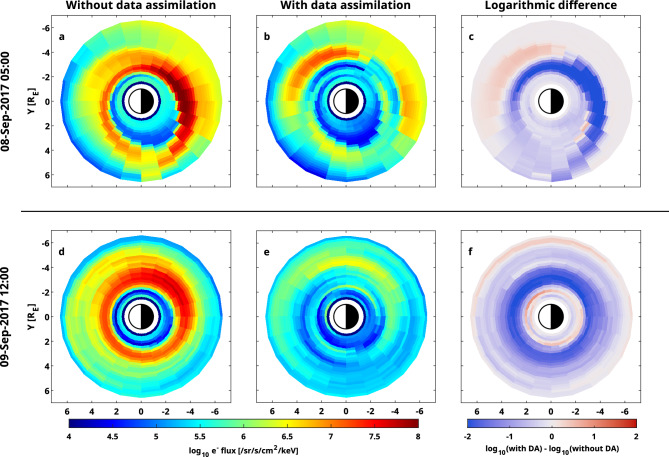
Global electron flux maps both with and without data assimilation for different times. (**a**) 10 keV electron flux with 30° pitch angle predicted by the standalone model for 05:00 September 8, 2017. (**b**) Electron flux predicted by the data-assimilative model (**c**) Logarithmic difference between the electron flux given by the two models. (**d**–**f**) Same format as (**a**–**c**) but for 12:00 September 9, 2017.

The global effect of the assimilation process can be seen in Fig. 3. While at the beginning of the storm, the simulations with and without data assimilation do not show much difference, they start to diverge soon, as the simulation without data assimilation predicts very high flux values around L = 3, which is corrected in the data-assimilative simulation. In general, the standalone model predicts higher flux values at low L compared to the data-assimilative run. The assimilation of Arase measurements on the dusk side truly has a global effect on the simulation and introduces differences of up to 2.5 orders of magnitude.

**Figure 4:**
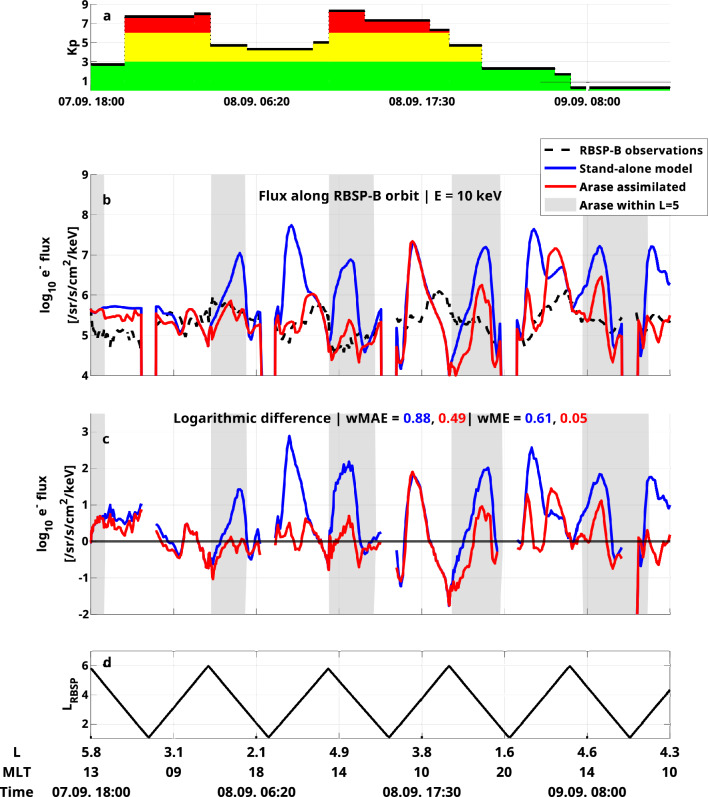
Validation against electron flux measurements taken by RBSP-B. (**a**) Kp time series (**b**) Observation and simulation results with and without data assimilation for 10 keV and 30° equatorial pitch angle along the RBSP-B orbit. The gray shaded area indicates times, when Arase provided measurements for data assimilation. (**c**) Logarithmic difference between observations and simulation results for 10 keV. Computed metrics are also shown for both simulations (see text for details). (**d**) L shell measured by the RBSP-B satellite.

We validate our data assimilation approach by comparing it against local satellite measurements, which are not used for assimilation. Figure 4 displays the VERB-CS results for 10 keV electron flux along the RBSP-B orbit both with and without data assimilation. The simulation results for 30 and 50 keV are presented in the supplementary material (see Figs. S3 and S4). Following our previous work^39^, we are showing data on a modified x-axis, which linearizes the satellite trajectories in order to remove the visual bias towards higher L shells. This bias would appear due to the satellite spending most of its time above L = 4.5. Without the help of data assimilation, VERB-CS overestimates the electron flux values up to three orders of magnitude at radial distances around 3 $R_E$. This overestimation can be explained by too long electron lifetimes in the pre-midnight sector, as demonstrated in previous work^40^. To quantify the discrepancies between measurements and simulation results, we calculate a weighted mean absolute logarithmic difference (wMAE) to describe the accuracy of the simulation, while the bias is described by the weighted mean logarithmic difference (wME). To avoid a bias towards large radial distances, we weigh each point of the measurements with the occurrence rate of the measured L shell, using L bins with a width of 0.25^39^. The wMAE of the standalone model is 0.88, while the wME is 0.61, showing a significant overestimation of the satellite observations.

Assimilating Arase data into the initial simulation substantially improves simulation results. The large overestimation at low radial distances is greatly reduced, if not completely removed for most trajectories of the satellite. The correction of too high flux (compared to observations) can also be seen in Fig. 3, where a large negative innovation was introduced by the Kalman filter at 3 < L < 4. However, for one trajectory happening around September 8, 20:00, the simulation results did not improve after assimilating Arase data. This is due to Arase measuring particles beyond L = 5 during that time (see gray-shaded regions), causing the data to not actually be assimilated. Overall, the wMAE is reduced to 0.61, while the wME is reduced to 0.05. We conclude that the bias of the simulation was almost removed completely as indicated by the change in the wME, while the accuracy also improved considerably.

## Discussion

**Figure 5:**
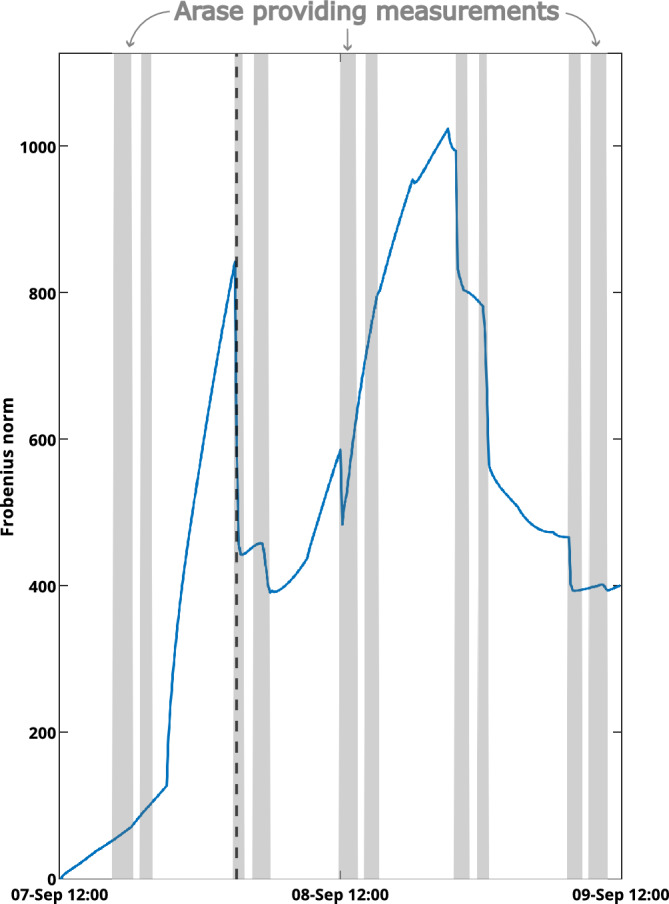
Evolution of the Frobenius norm of the covariance matrix for $\mu = 0.5 \hbox {MeVG}^{-1}$ and $\hbox {K} = 0.6 \hbox {G}^{1/2}\hbox {R}_{\textrm{E}}$. The vertical dashed black line indicates the time step seen in Fig. 6.

**Figure 6:**
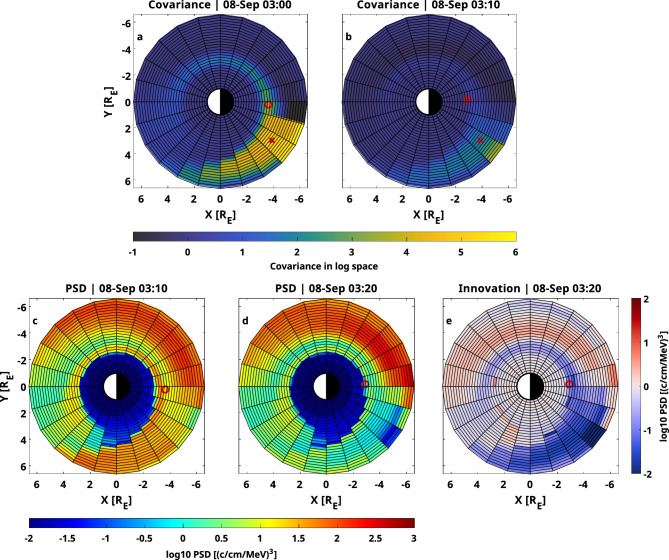
Covariances and corresponding PSD output and innovation vector for two consecutive time steps and $\mu = 0.5 \, \hbox {MeVG}^{-1}$ and $\hbox {K} = 0.6 \, \hbox {G}^{1/2}\hbox {R}_{\textrm{E}}$. (**a**) Covariances in log space at time step one originating from the grid point marked by the red cross. The red circle shows the position of the Arase satellite. (**b**) Same as (**a**), but for the next time step. (**c**) PSD output resulting from the covariance in (**a**). (**d**) PSD output resulting from the covariance in (**b**). (**e**) Difference between both PSD outputs.

In this work, we have shown how assimilating single-point measurements using a standard log-normal Kalman filter can affect electron ring current simulations in a global manner. The most critical error of the standalone physics-based model, an overestimation around radial distances of 3 $R_E$ throughout the storm, was eliminated for most satellite trajectories. Even though the assimilated satellite resides on the dusk side, comparison with the validation satellite residing on the dayside demonstrates a global improvement of the simulation. This is partly due to the growing covariance matrix when no measurements are available, as highlighted in the Methods section. The Kalman filter correlates grid points along electron drift trajectories, thereby connecting grid points from the nightside all the way to the dayside (see Fig. 6). Once measurements are available again, the ring current simulation is globally corrected by the Kalman filter and the correlations are reduced (see Fig. 5). Although it seems like this behavior of a growing covariance matrix is desirable for our case of application, where satellite data may be sparse in time, it can also lead to problems, as the large correlations predicted by the Kalman filter may not be realistic. As the loss term due to wave-particle interactions has to be omitted in order to linearize the model, the Kalman filter uses an incorrect representation of our model to predict the covariances. Additionally, the convection problem within the Kalman filter is solved using a 1st order method leading to a large amount of numerical diffusion. This means that not only will grid points along the particle’s drift trajectory be correlated, all grid points linked through numerical diffusion will be correlated as well. These factors potentially lead to unrealistic covariance matrices, and their resulting influences on the electron flux are difficult to predict. In rare cases (see Fig. 4 around 22:00 on September 8) we observe that the assimilative model performs worse compared to the standalone model when compared to the measurements from the validation satellite. This could be due to an incorrect representation of the covariance matrix or due to errors in the calculation of the particles’ drift trajectories themselves. The issues introduced by having to use a linearized model can be overcome with more sophisticated filtering techniques. Ensemble Kalman filters^18^ can be used with non-linear models, while also having the advantage of requiring less memory for computations compared to the standard Kalman filter. This filter technique has been used in previous studies with great success for assimilating satellite measurements into three-dimensional radiation belt models^20^. The main disadvantage of the ensemble Kalman filter is the high computational cost, necessary to calculate enough ensemble members to ensure convergence. Hower, this study already demonstrates the great potential of the log-normal Kalman filter as a suitable algorithm for operational data-assimilative ring current simulations.

Another limitation of the method applied in this work is the limited space and pitch angle coverage of the assimilated satellite. When Arase is not providing measurements for the Kalman filter, the simulation is not corrected, and overestimations persist in the resulting electron flux. Our results show that one satellite on a highly elliptical orbit is not enough to correct ring current simulations globally at all times during geomagnetic storms, as the satellite does not pass through the heart of the ring current frequently enough. One could use measurements from LEO satellites, which provide measurements of this region with a much higher cadence. However, they are in turn limited by solely measuring particles with small equatorial pitch angles due to their high magnetic latitude. We also encounter these limitations in this study, as we have to restrict our validation to equatorial pitch angles under 30°. One possible way to address this issue is to include the pitch angle diffusion term in the model, which allows information to travel across the pitch angle dimension as well. Another way for correcting high pitch angles using observations at LEO is extrapolating the measurements to higher pitch angles, potentially introducing inaccuracies. There are models already developed for this purpose, which could be used in future studies^41–45^. Ideally, for an operational model, one would want to use all available satellite missions simultaneously, to collect as much data as possible. In this case, the instruments would also have to be carefully intercalibrated, as their measurements can potentially be largely biased and in disagreement with each other^46,47^.

The impact of the data assimilation may also be highly sensitive to the observed MLT sector. In this study, we chose an event with an advantageous case, where the data-providing satellite covers the midnight region, where it can observe the injected electrons immediately before they drift around Earth. Assimilating data measured by the Van Allen Probes on the dayside also has a big effect on the electron flux on the nightside (see Supplementary Fig. S9). Validating against Arase measurements shows that the assimilation eliminates almost all the errors during the recovery phase of the storm. During the main phase of the storm, we are seeing mixed results and no clear conclusions can be made from this single experiment. Observing system experiments and observing system simulation experiments should be performed in future studies to determine the most effective location of satellites for data assimilation.

This study demonstrates the effectiveness of assimilating sparse satellite data into electron ring current simulations for correcting the global state of the simulation. When compared to measurements from an independent satellite flying in a different MLT sector, the accuracy of our model predictions has improved significantly, while the bias was almost completely eliminated. We have shown the great potential data assimilation holds for space weather applications, including operational ring current models.

## Methods

### The VERB-CS model

The four-dimensional Versatile Electron Radiation Belt code (VERB-4D)^48^ describes the evolution of phase space density with a convective-diffusive equation in MLT, radial distance *R*, and the two modified adiabatic invariants *V* and *K*^49^:

1 $$\begin{aligned} K=\frac{J}{\sqrt{8m_0\mu }}\quad \text {and}\quad V=\mu (K+0.5)^2, \end{aligned}$$

where $\mu$ and *J* represent the first and second adiabatic invariants^50^, and $m_0$ is the rest mass of an electron. VERB-4D has been extensively used for simulating radiation belts^48,51^, plasmasphere^38,52^, and ring current dynamics^53^.

In this work, we use a simplified version of VERB-4D, based on realistic assumptions on the dynamics of particles in the target energy range (< 50 keV). As energy diffusion acts on larger time scales compared to pitch angle scattering^54^, it can be considered insignificant for this application. Since mixed diffusion terms can be neglected as well, this allows us to model pitch angle scattering as a uniform decay of the pitch angle distribution with the lowest normal mode^55,56^. The associated eigenvalue can be interpreted as the lifetime of the electrons and it can be derived from diffusion coefficients. The diffusion in $L*$ is neglected as well, as particles below 50 keV are most likely on open drift paths during the main phase of a geomagnetic storm, causing the $L*$ parameter of these particles to be undefined.

The resulting model is called VERB-CS (Convection Simplified)^53^, which solves the following equation in each time step:

2 $$\begin{aligned} \frac{\partial f}{\partial t}= - \langle v_\varphi \rangle \frac{\partial f}{\partial \varphi } - \langle v_R \rangle \frac{\partial f}{\partial R} - \frac{f}{\tau _{wave}}, \end{aligned}$$

where *f* represents the phase space density, $\langle v_\varphi \rangle$ and $\langle v_R \rangle$ are the bounce-averaged drift velocities in MLT and *R*, and $\tau _{wave}$ represents the lifetimes of electrons associated with wave-particle interactions caused by hiss and chorus waves. Drift velocities are calculated using a combination of the Volland-Stern electric field^57,58^ with the Maynard-Chen Kp parameterization^59^ and a Kp-dependent subauroral polarization stream module^60^, while utilizing the T89 magnetic field model^61^. Outside the plasmapause location, electron lifetimes due to chorus wave scattering^62^ are applied, while inside lifetimes due to hiss wave scattering^63^ are used. The plasmapause location is determined using the Kp-dependent model proposed by Carpenter and Anderson^64^. We do not model the losses arising from magnetopause shadowing, as these losses are negligible without radial diffusion.

The description of Eq. (2) would not be complete without initial and boundary conditions. The initial condition for the simulation is based on the last full pass of the RBSP-B satellite before the simulations begin, assuming MLT-isotropy. The flux at the radial boundary at geosynchronous orbit is described by the statistical mean from the Kp-dependent Denton model^65^, while the flux at the radial boundary at Earth’s surface is set to 0.

The numerical grid is set up with 25 grid points for the MLT dimension and 29 points for *R*, covering the range from 1 to 6.6 $R_E$. This results in a coarser spatial grid compared to previous studies^40,53^, which is necessary to limit the memory requirement of the Kalman filter. The *V* and *K* dimensions are divided into 21 and 20 points, respectively, on a logarithmic grid, ensuring that the target energy and pitch angle ranges are defined for all radial distances. The time step of the simulation is 10 min.

### Data preparation

In this work, we use release 04 of the HOPE level 3 data and version v01_01 of the MEPe level 3 data. The data is further processed by binning the measurements in 5 min bins using the median while ignoring NaN values. The T04s magnetic field model^31^ is used for mapping the satellite locations along the field lines to the magnetic equator and calculating the observed equatorial pitch angles. As VERB-CS is calculating phase space density, flux measurements have to be converted to phase space density before they can be assimilated. We utilize the T04s magnetic field model to convert Arase flux measurements into phase space density and calculate associated modified adiabatic invariants. For every time step, all measurements between that time step and the previous one are considered for data assimilation. The measurements are binned in space, and linearly interpolated in invariant space to match the model grid. Both operations are performed using the logarithm of phase space density and adiabatic invariants.

### The log-normal Kalman filter

The motivation behind the log-normal Kalman filter is that phase space density is a strictly positive quantity, hence it is desired, that a data assimilation algorithm ensures this positivity^21,22^. This can be realized by modelling the natural logarithm of phase space density:

3 $$\begin{aligned} g = \log {f}, \end{aligned}$$

which can be used to transform Eq. (2):

4 $$\begin{aligned} \frac{\partial g}{\partial t}= - \langle v_\varphi \rangle \frac{\partial g}{\partial \varphi } - \langle v_R \rangle \frac{\partial g}{\partial R} - \frac{1}{\tau _{wave}}. \end{aligned}$$

The Kalman filter assumes that the error distributions of both the measurements and the model are Gaussian. As the phase space density changes by orders of magnitudes, the error distributions are more Gaussian in log space than in linear space. This further motivates the usage of the logarithmic formulation for the filtering problem.

The Kalman filter is based on the assumption of a linear dynamical system described in standard form by:

5 $$\begin{aligned} x^{t+1} = A x^{t}, \end{aligned}$$

where *x* is the system state and *A* is a constant matrix describing the evolution of the system. Equation 4 does not represent such a linear dynamical system due to the loss term and cannot be written in standard form. This issue can be solved by dropping the loss term, resulting in the final model equation used for the filtering problem, which can be written in matrix form when discretized in time and space:

6 $$\begin{aligned} \frac{\partial g}{\partial t}= - \langle v_\varphi \rangle \frac{\partial g}{\partial \varphi } - \langle v_R \rangle \frac{\partial g}{\partial R}. \end{aligned}$$

As the loss by wave-particle interactions is a crucial process for the electron ring current^39^, this represents a limitation of the standard Kalman filter. It would be possible to keep the loss term if the linear Eq. (2) were used inside the filter, however this adjustment introduces the downsides of negative values and non-gaussian error distributions. Testing this approach gave unsatisfying results in the early stage of this study, and therefore was no longer considered. Next, the two-dimensional advection term has to be written in matrix form. In VERB-4D and VERB-CS, the advection is solved using an explicit 9th order upwind scheme^66^ coupled with a flux limiter and a discriminator^67^, to preserve large gradients and local maxima^68^. Since the flux limiter and discriminator are non-linear operations, they cannot be written in matrix form. Thus, we have to fall back on a simple explicit first-order upwind scheme for discretizing the advection terms, as higher order terms would result in unphysical oscillations^66^. Solving the two-dimensional advection equation requires the following stability condition:

7 $$\begin{aligned} c_\varphi + c_R \le 1, \quad c_\varphi = \langle v_\varphi \rangle \frac{\Delta t}{\Delta \varphi }, \quad c_R = \langle v_R \rangle \frac{\Delta t}{\Delta R}, \end{aligned}$$

where $c_\varphi$ and $c_R$ are the Courant numbers, $\Delta \varphi$ and $\Delta R$ are grid resolutions, and $\Delta t$ is the time step, which is determined by the inequality in Eq. (7). We want to emphasize that the predicted state of the system is calculated using the non-linear model given by Eq. (2) and the 9th order scheme. Only the covariances are predicted using the linear model described above.

Regarding the equations of the log-normal Kalman filter, we refer to a previous study, where the algorithm is presented in great detail^14^. Following previous work^14,22,69^, we assume uncorrelated white noise using $Q = R = \text {diag}(\log {1 + \alpha })$, where *Q* are the covariances of the process noise, *R* are the covariances of the observation noise, and $\alpha = 0.5$ is the assumed variance. We tested different values of $\alpha$ and found that the assimilated simulation results are not sensitive to this parameter (see Supplementary Fig. S5).

### Evolution of covariances

Another important detail of our data assimilation approach is that we are updating the covariance matrix in every assimilation step, even when no measurements are available. This means evolving the covariance matrix using the linearized model matrix and adding variances to each grid point [see Eq. (15) in^14^]. Since the model matrix represents physical advection, the variance of a grid point will be moved in the spatial domain, and now represents a covariance between the resulting grid point and the original one. By repeating this process, grid points far away from each other will be correlated if they are connected by the drift trajectories of electrons.

In this study, we assimilate Arase measurements, which are very sparse in time for this event at our chosen equatorial pitch angle (see Fig. 1). Hence, it is interesting to see how the covariance matrix evolves over time. In Fig. 5, the Frobenius norm of the covariance matrix for a single adiabatic invariants pair is plotted over the simulation time. When Arase does not provide measurements for the Kalman filter, due to it measuring electrons with too low equatorial pitch angles, the covariances grow rapidly, resulting in large Frobenius norms. When measurements are available, the filter becomes more confident in the predictions, as the Frobenius norm of the covariance matrix is quickly shrinking again.

It is useful to visualize the covariances between a single grid point and all other grid points for a single adiabatic invariant pair to see the physical meaning behind these uncertainties (see Fig. 6). High covariances can be seen along the electron drift trajectory crossing the grid point under investigation. As data to assimilate has not been available for a long time, the covariances have grown towards the dayside, linking the whole spatial domain. A single-point measurement, when assimilated into the model, will now have an immediate global effect on the simulation, as almost all grid points will be correlated with the measurement. In this case, the Arase measurements taken on the nightside result in an innovation of up to 2 orders of magnitude, and affect the simulation globally.

We also test the importance of updating the covariance matrix during every time step regardless of the availability of measurements, by only updating the matrix, when Arase is actually providing measurements to assimilate. The resulting figures are provided in the supplementary material (see Figs. S6, S7 and S8), and show that this approach is not capable of correcting the global state of the simulation.

### Supplementary Information

Supplementary Figures.

## Data Availability

All HOPE RBSP-ECT data are publicly available at the website https://rbsp-ect.newmexicoconsortium.org/data_pub/rbspb/hope/level3/pitchangle/. MEPe L3 electron flux data is available at the website https://ergsc.isee.nagoya-u.ac.jp/data/ergsc/satellite/erg/mepe/. Dst and Kp values are from the NASA OMNIWeb data explorer, accessible at https://omniweb.gsfc.nasa.gov/form/dx1.html.
