# Supplementary material for: Global validation of data-assimilative electron ring current nowcast for space weather applications
Source: Sci Rep. 2024 Jan 28;14:2327. doi: 10.1038/s41598-024-52187-0 (PMC10822866; doi:10.1038/s41598-024-52187-0)
Supplement: Supplementary file 1 — Supplementary Figures. [file 41598_2024_52187_MOESM1_ESM.pdf]

Supplementary Material for Article titled:

'Global Validation of Data-Assimilative Electron Ring Current Nowcast for Space Weather Applications'

Bernhard Haas[1,2,*], Yuri Y. Shprits[1,2,3], Michael Wutzig[1], Mátyás Szabó-Roberts[1], Marina García Peñaranda[1,2], Angelica M. Castillo Tibocha[1,2], Julia Himmelsbach[1], Dedong Wang[1], Yoshizumi Miyoshi[4], Satoshi Kasahara[5], Kunihiro Keika[5], Shoichiro Yokota[6], Iku Shinohara[7], and Nana Higashio[7]

1 GFZ German Research Centre for Geosciences, Helmholtz Centre Potsdam, Potsdam, Germany

2 Institute of Physics and Astronomy, University of Potsdam, Potsdam, Germany

3 Department of the Earth, Planetary and Space Sciences, University of California, Los Angeles, CA, USA

4 ISEE, Nagoya University, Nagoya, Japan

5 School of Science, University of Tokyo, Tokyo, Japan

6 Osaka University, Toyonaka, Japan

7 Japanese Aerospace Exploration Agency, Tokyo, Japan

*bhaas@gfz-potsdam.de

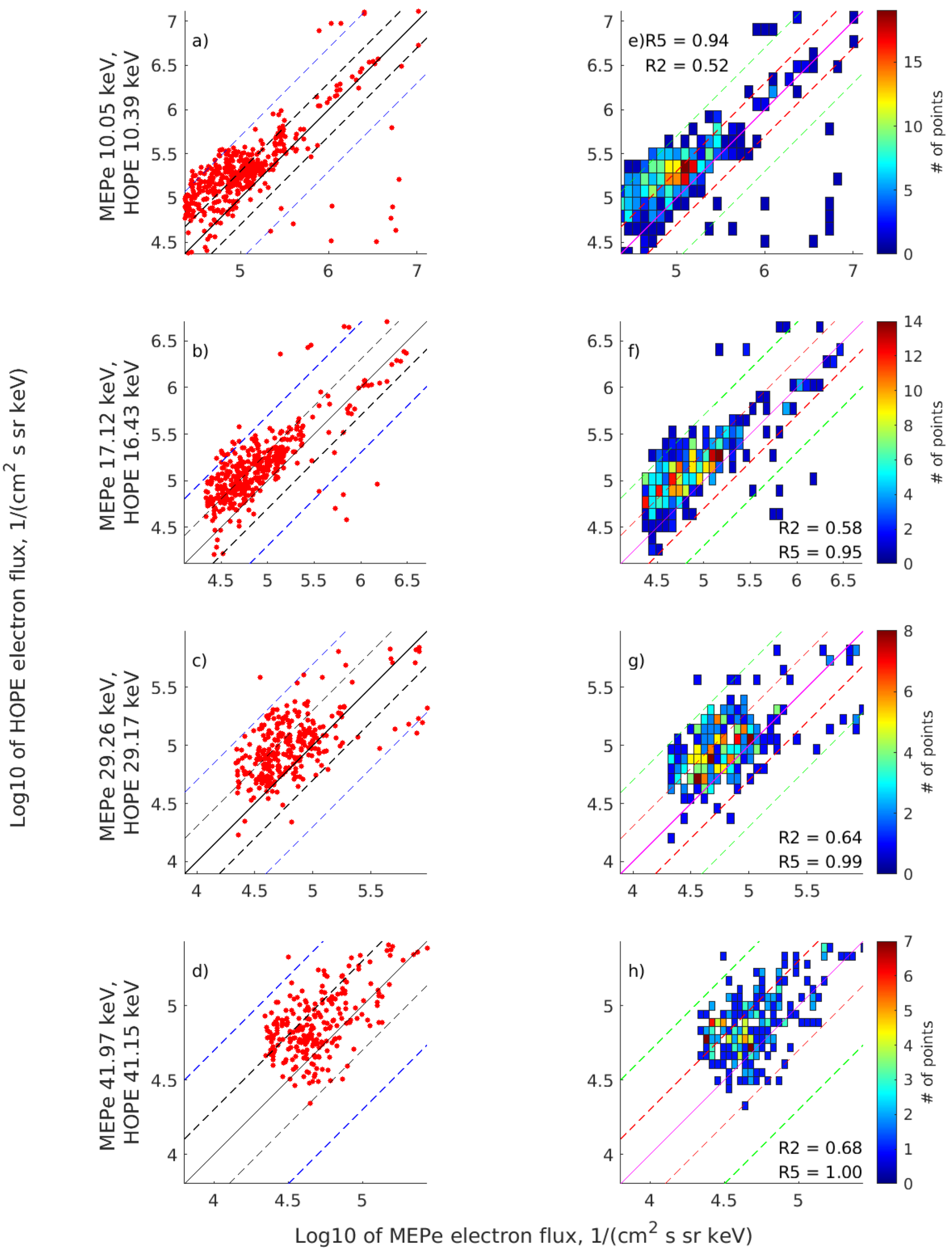

Figure S1: Comparisons between MEPe and RBSP-A HOPE pitch angle sorted electron flux at magnetic conjunctions. Conjunctions are defined as the the two satellites being within +-1 hour in MLT, +-0.2 in Lm, and +-10 degrees in maximum equatorial pitch angle observed, for the same 5 minute interval. Each row represents a different set of energies. Since several of the energy channels of the two instruments are very close to each other, we chose not to interpolate either set of measurements in energy. Additionally, we implemented a cutoff at the 10-count level for both sets of measurements in order to avoid signal-to-noise and noise-to-noise comparisons. We used the provided 10-count level flag for HOPE and the provided g-factor for MEPe. Panels a)-c) show the fluxes at these conjunctions, while panels e)-h) show a histogram of the observations to illustrate how they are distributed. On all panels, the solid black line indicates one-to-one correspondence, the dashed black line denotes agreement within a factor of two, and the blue dashed line denotes agreement within a factor of 5. Panels e)-h) also include numerical values denoted by R2 and R5, which stand for fraction of observations agreeing within a factor of 2 and 5, respectively.

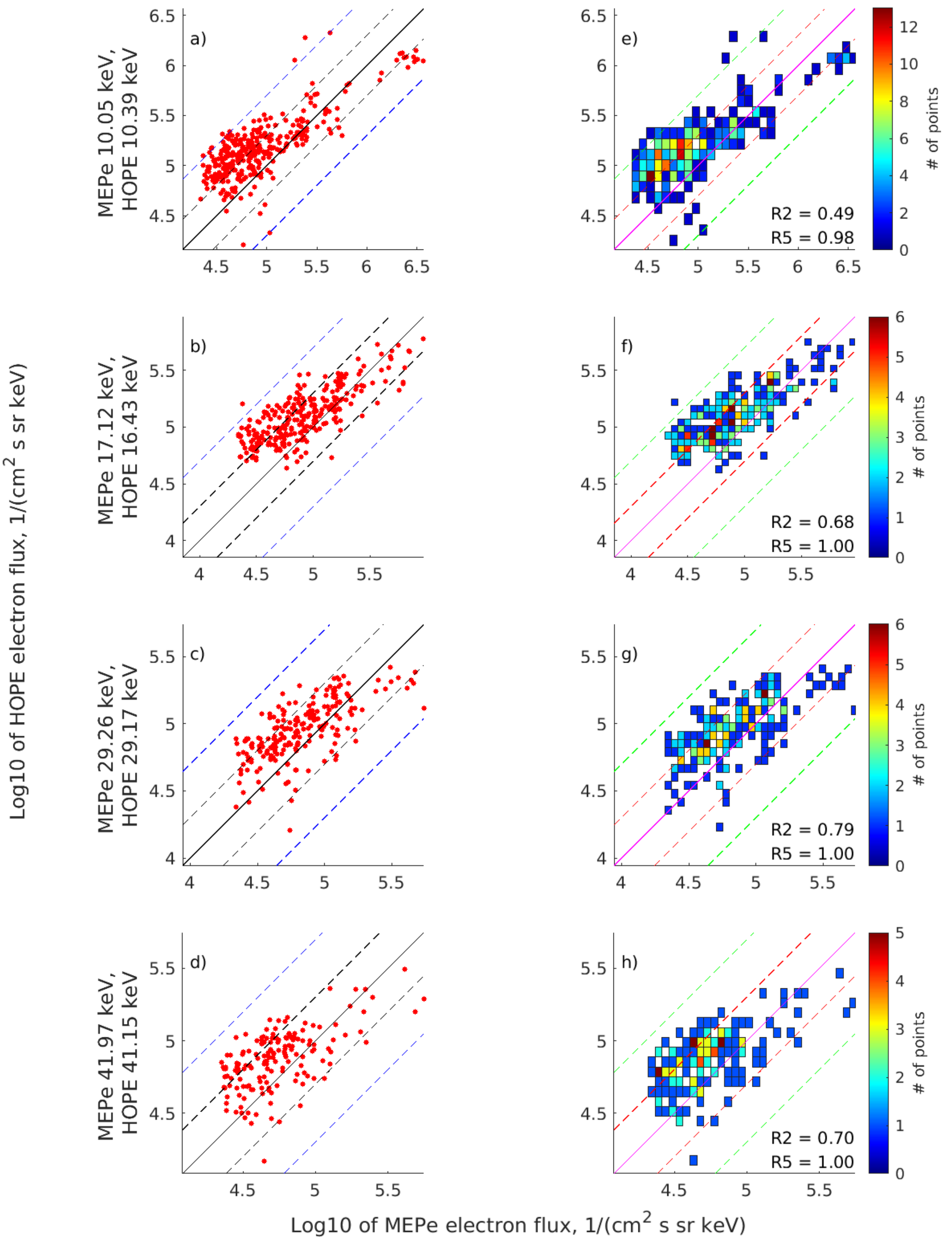

Figure S2: Same format as Figure S1, but now for RBSP-B.

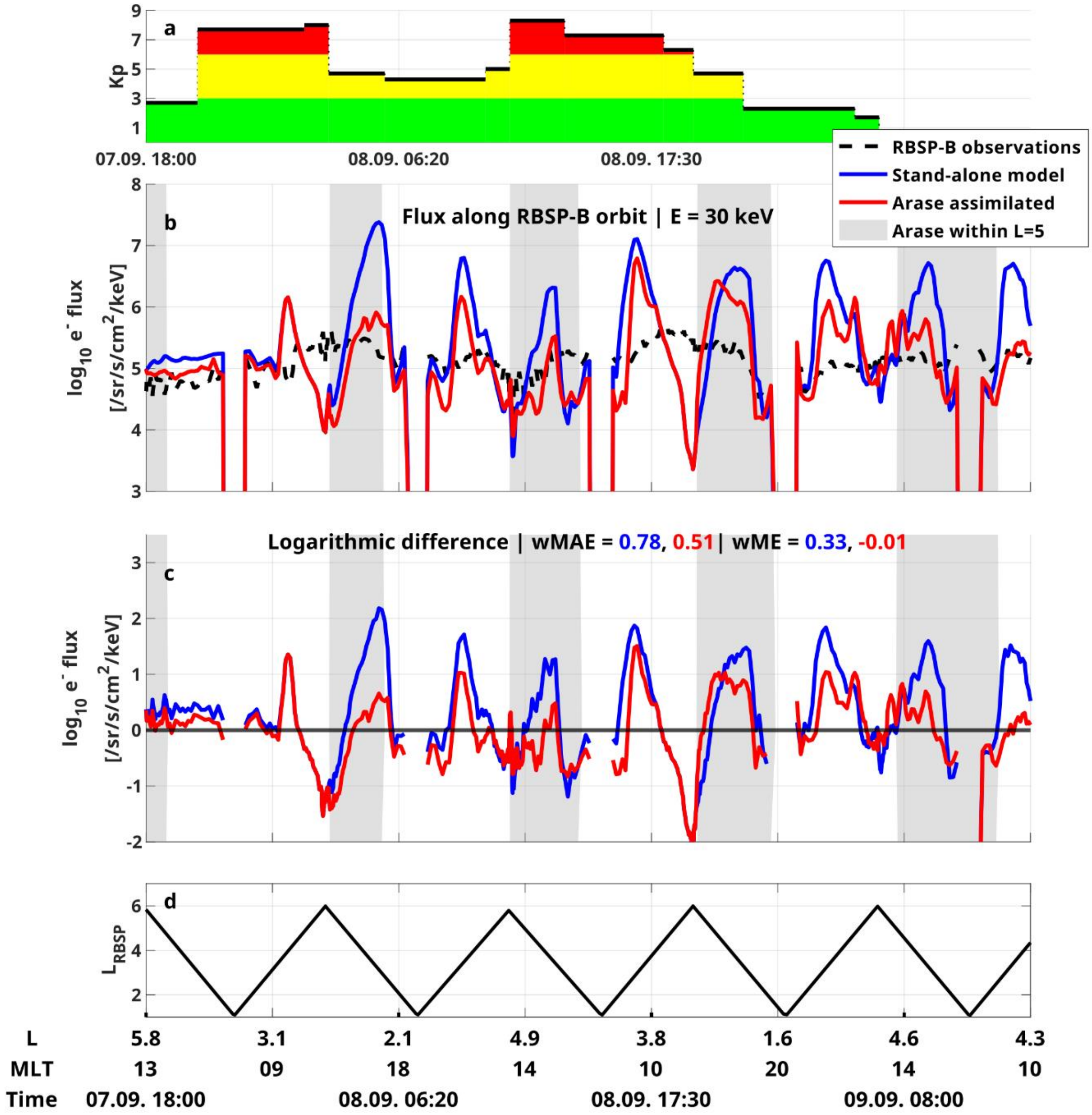

Figure S3: Same format as Figure 4 from the main manuscript, but for 30 keV electron flux.

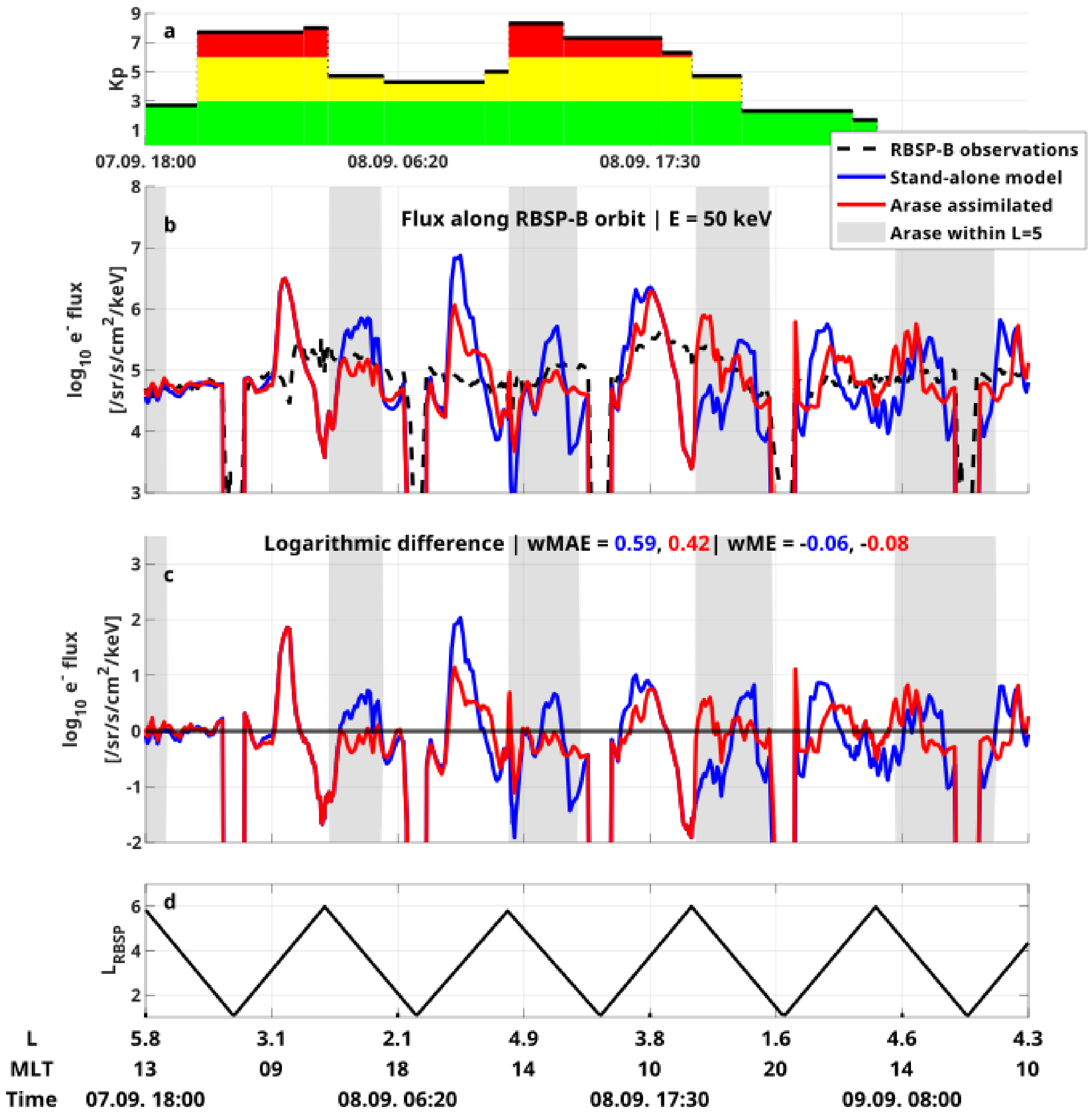

Figure S4: Same format as Figure 4 from the main manuscript, but for 50 keV electron flux.

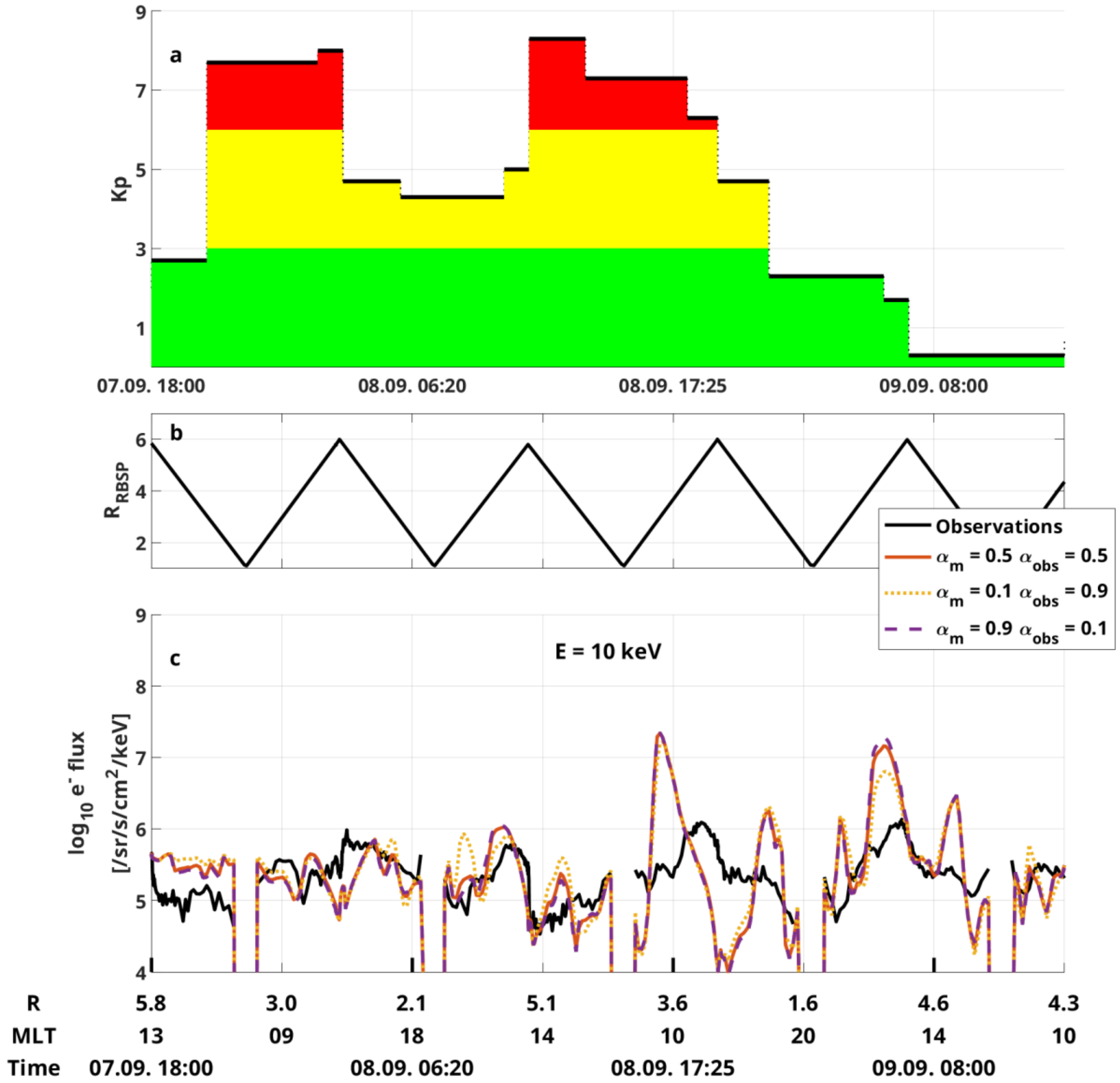

Figure S5: Sensitivity analyses of data assimilative simulation results regarding the assumed variances (α_m = model variance, α_{obs} = observation variance). We conclude that the assumption of the variance is an insignificant parameter.

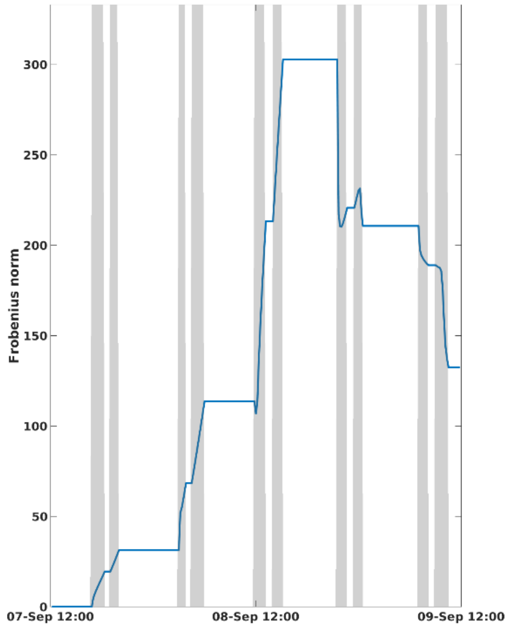

Figure S6: Same format as Figure 5 from the main manuscript, but now the covariance matrix is not updated, if no measurements are available. One can see how the maximum of the Frobenius is much lower compared to the setup of constantly updating covariance matrix.

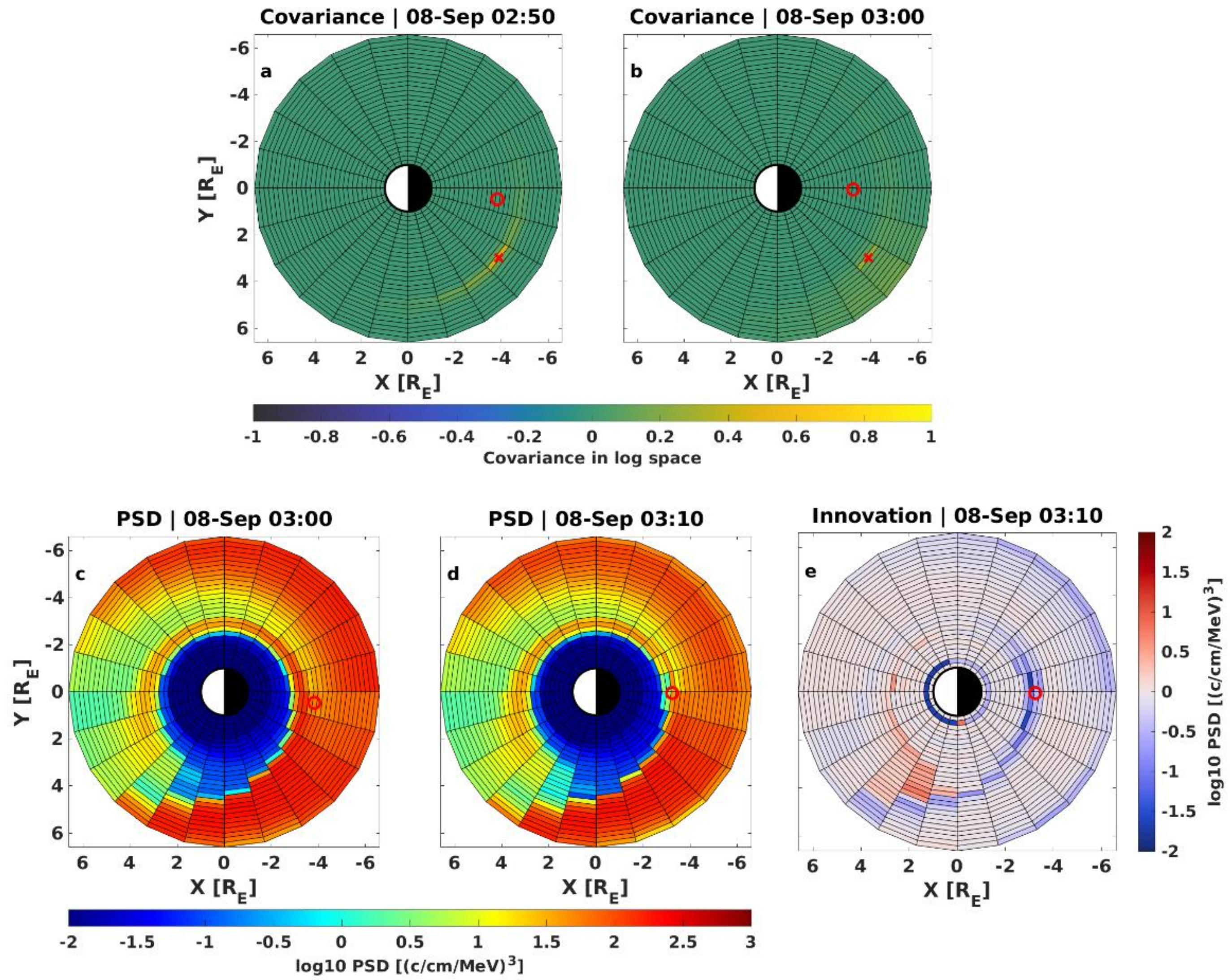

Figure S7: Same format as Figure 6 from the main manuscript, but now the covariance matrix is not updated, if no measurements are available. The covariances are much lower and do not cover the whole spatial grid. Assimilating the measurements of Arase now have a much smaller influence on the global state of the simulation.

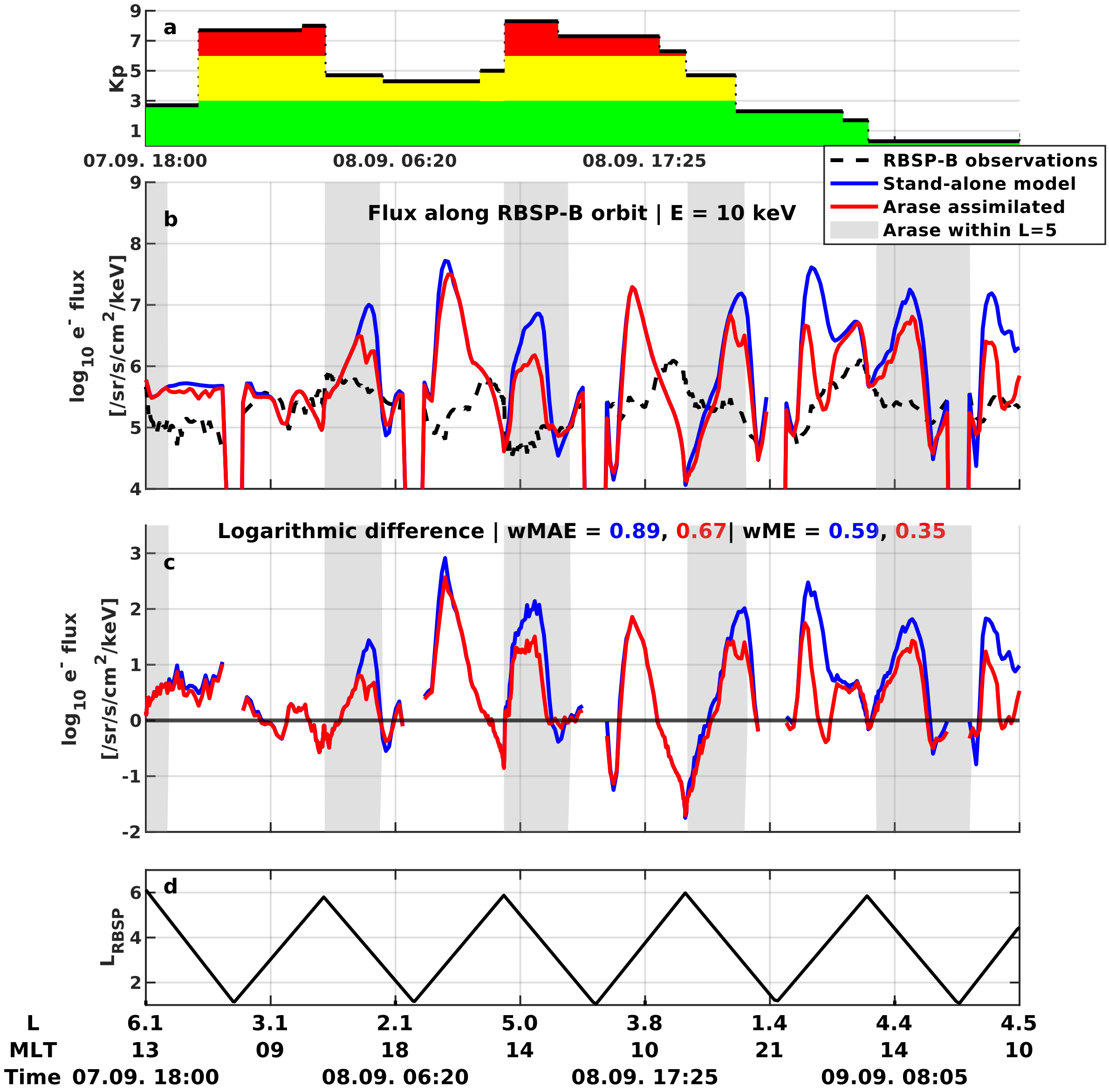

Figure S8: Same format as Figure 4 from the main manuscript, but now the covariance matrix is not updated, if no measurements are available. The data-assimilative model still performs better, but not as well as the model with constantly updated covariance matrices.

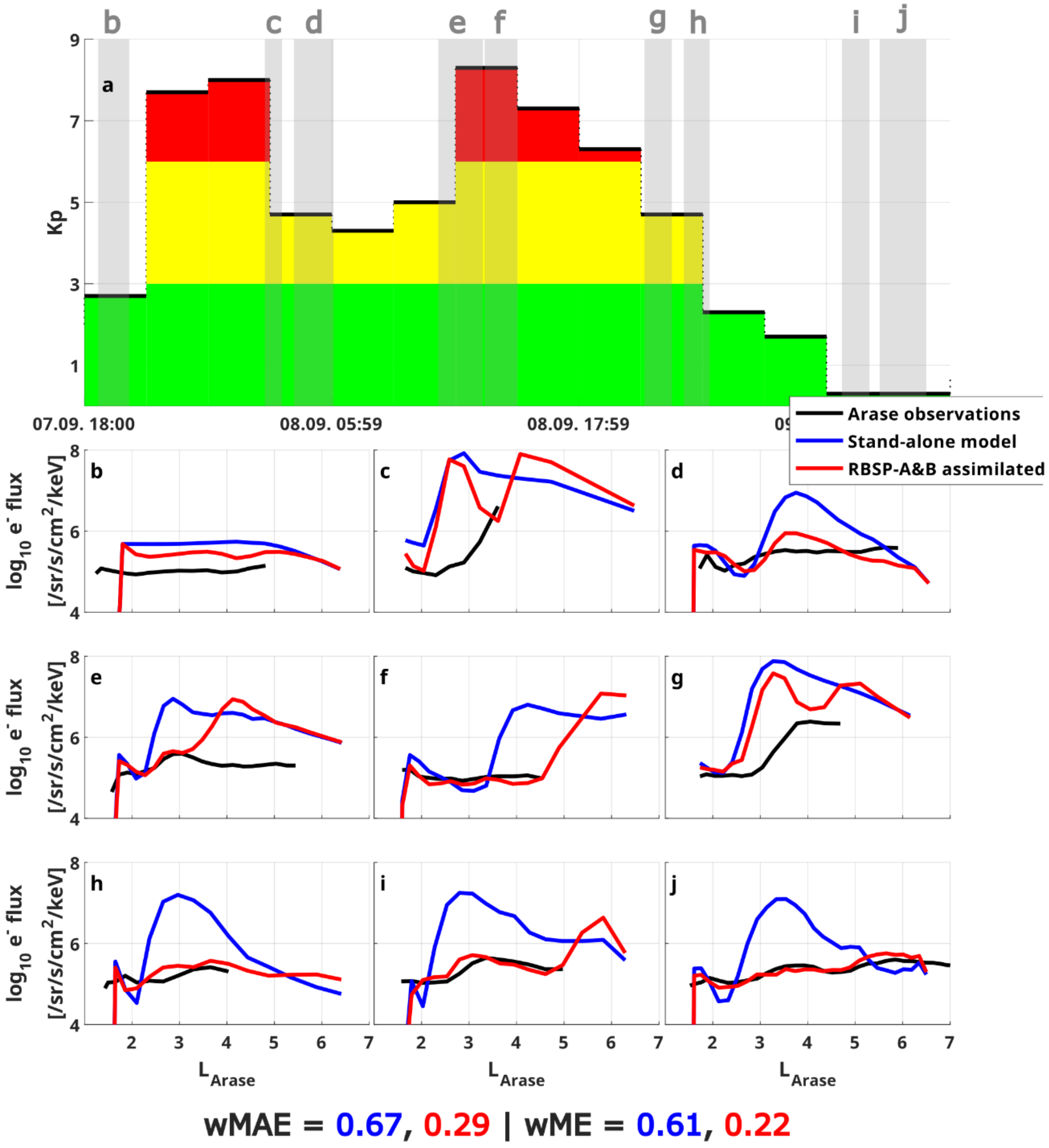

Figure S9: Assimilation of RBSP-A&B and validation against Arase. The grey areas in the Kp plot (Panel a) show the time period, when Arase is observing particles within geosynchronous orbit. The measured and simulated flux along each inbound and outbound trajectory is shown as a single panel (Panels b-j). The letters above the grey areas are corresponding to the individual trajectory panels. We see great improvement during the recovery phase of the storm (Panels h-j), while we are getting mixed results during the main phase of the storm (Panels c-g).
